# Social and relational identification as determinants of care workers’ motivation and well-being

**DOI:** 10.3389/fpsyg.2015.01460

**Published:** 2015-10-15

**Authors:** Kirstien Bjerregaard, S. Alexander Haslam, Thomas Morton, Michelle K. Ryan

**Affiliations:** ^1^School of Psychology, University of ExeterExeter, UK; ^2^School of Psychology, University of Queensland, BrisbaneQLD, Australia

**Keywords:** care work, motivation, social identity, organizational identity, relational identity

## Abstract

A growing body of research in the field of health and social care indicates that the quality of the relationship between the person giving care and the person receiving it contributes significantly to the motivation and well-being of both. This paper examines how care workers’ motivation is shaped by their social and relational identification at work. Survey findings at two time points (T1, *N* = 643; T2, *N* = 1274) show that care workers’ motivation increases to the extent that incentives, the working context (of residential vs. domiciliary care), and the professionalization process (of acquiring vs. not acquiring a qualification) serve to build and maintain meaningful identities within the organization. In this context care workers attach greatest importance to their relational identity with clients and the more they perceive this as congruent with their organizational identity the more motivated they are. Implications are discussed with regard to the need to develop and sustain a professional and compassionate workforce that is able to meet the needs of an aging society.

## Introduction

In the UK as elsewhere across the industrialized world, the rapidly aging population has led to a dramatic increase in the size of the adult social care workforce and this is increasingly recognized as an important social and economic resource (Centre for Workforce Intelligence, 2011; Care Quality Commission, 2012; Department of Health, 2012; Skills for Care, 2012, 2013c). In the UK, for example, the direct economic value of the adult social care sector is estimated to be worth more than £20 billion per year. The sector employees over 1.5 million people — which is more than either the construction industry or the public administration and defense sectors (Skills for Care, 2013b). Moreover, this sector is predicted to grow to employ between 2.1 and 3.1 billion people by 2025 (Centre for Workforce Intelligence, 2011). Nevertheless, with some notable exceptions, very little research has looked in any depth at the substrates of care workers’ motivation or at what sustains their capacity to deliver professional and compassionate care (Help the Aged, 2007; Lucas et al., 2008; Hussein et al., 2010; Adamson et al., 2012).

The conundrum of care worker motivation is exemplified by the fact that in spite of their low status and poor financial reward, domiciliary, and care home workers report high levels of job satisfaction, pride and well-being (Cameron and Moss, 2007; Skills for Care, 2007; Lucas et al., 2009; Hussein et al., 2010). This is largely attributed to care workers’ intrinsic satisfaction with their work. Indeed, in this context financial and altruistic incentives are frequently cited as polarized aspects of carers’ motivation — such that the “virtuous reward” of caring (Heyes, 2005, p. 1) is often viewed as compensating for, and even justifying, the lack of financial reward (Heyes, 2005; Equality and Human Rights Commission, 2012).

More recently, though, there have been calls for theories to challenge the dominant social discourse that quality of caring is shaped by whether it is done ‘for love’ or ‘for money,’ and to instead consider how virtuous and financial reward might be integrated aspects of care workers motivation (Nelson and Folbre, 2006; Lepore, 2008; Hussein et al., 2010; Weicht, unpublished). However, as noted in a review of the role of reward and incentives in adult social care in England (conducted on behalf of Skills for Care, 2007), although at a common-sense level one might conclude that there are links between pay, other incentives, and the recruitment and retention of care workers, to date very little work has explored these linkages (Lucas et al., 2008).

In what work there has been on this topic, care workers’ motivation has consistently been found to be associated with their relationships with clients (Skills for Care, 2007; Lucas et al., 2009; Maben et al., 2012; Adams and Sharp, 2013; Bjerregaard et al., in press a). Moreover, the quality of this relationship is also perceived (by clients and their families) to be indicative of the quality of care (Bowers et al., 2001; Wilson et al., 2009). The centrality of this relationship to the motivation and well-being of care workers and clients alike has also underpinned the drive for a relationship-centered approach to health and social care (Beach and Inui, 2006; Nolan et al., 2006) and the creation of compassionate care-work cultures (Adamson et al., 2012; Department of Health, 2012; Onyett, 2012; Dewar and Nolan, 2013). As yet, though, there has been little consideration of the psychological processes through which these work relationships contribute to care workers’ motivation.

The context in which the carers work is also likely to shape their motivation. Long-term adult care usually takes place either in residential and nursing homes or in the form of domiciliary care in people’s homes. In the former, carers work together to look after a number of residents, whereas in the latter carers typically visit people in their own homes and work independently. The two domains thus require different working styles and often attract people seeking different things from their work (Care Quality Commission, 2012; Bjerregaard et al., in press a). Accordingly, the implications of working in these different domains for motivation also needs to be taken into account when considering how best to create and sustain compassionate care organizational cultures.

In both spheres of activity, the ongoing professionalization of the sector over the last decade, in the UK and elsewhere, has resulted in the establishment of standards for workforce training and qualifications, the regulation of care practices, and the accountability of the sector to a professional body. Many discussions around this issue emphasize the importance of employee training and qualifications (e.g., Department of Health, 2006, 2012; Wild et al., 2010) and studies have found that care workers themselves identify training and development as important determinants of their work motivation (Wild et al., 2010; Skills for Care, 2013a,c). Again, though, very little, if any, research has examined whether and how training relates to the other motivational factors that sustain workers in the care sector. It is therefore pertinent to examine whether care workers’ pursuit and acquisition of qualifications enhances their motivation at work, and how this relates to other psychological outcomes such as well-being.

### Understanding Care Workers’ Motivation

As noted above, mainstream accounts of employee motivation foreground individualistic considerations and argue that work motivation is driven primarily by personal self-interest or the trade-off between money and altruistic reward. Accounts that go beyond a simple focus on economic (vs. other) reward nonetheless also theorize motivation as largely the outcome of personal factors. For example, *self-determination theory* (SDT; Ryan and Deci, 2000; Gagné and Deci, 2005) distinguishes between different types of motivation on a continuum between extrinsic controlled, “amotivation (which is wholly lacking in self-determination), to intrinsic [autonomous] motivation, which is invariantly, self-determined” (Gagné and Deci, 2005, p. 335). Pertinent to this distinction, a key finding is that incentives that activate extrinsic controlled motivation (e.g., those which set financial or processing targets) can actually erode intrinsic motivations and individual satisfaction (e.g., Deci, 1972). Indeed, on the basis of this argument, Heyes (2005) proposes an economic model in which “a badly paid nurse is a good nurse” (p. 1). At the same time, individuals are understood to differ in the degree to which behavior is externally regulated vs. integrated into the personal self, and this is also seen to vary as a function of context. Nevertheless, theorizing has yet to fully account for the psychological process through which this takes place (Greguras and Diefendorff, 2009; Kovjanic et al., 2012).

In contrast to these individually oriented accounts of motivation, the social identity approach offers an alternative framework which suggests that different levels of identity enactment might contribute to creating a (compassionate) working culture. This approach combines *social identity theory* (SIT; Tajfel and Turner, 1979) and *self-categorization theory* (SCT; Turner, 1985; Turner et al., 1987), and, alongside *organizational identity theory* (Ashforth and Mael, 1989; Ashforth et al., 2008), focuses on the ways in which a person’s work motivation is shaped both (a) by their sense of identification with different groups within an organization (Haslam et al., 2000; Van Knippenberg, 2000; Ellemers et al., 2004), and (b) by their identification with different role relationships (Sluss and Ashforth, 2008). Rather than defining the self in purely personal terms (as ‘I’ and ‘me’) this perspective argues that the self can also be defined in collective terms (as ‘we’ and ‘us’). So in addition to their idiosyncratic personal identity, a person’s self-concept is also understood to incorporate a range of social identities and role relational identities that become more or less salient depending on the fit and accessibility of the identity within a particular context (Oakes et al., 1994).

The definition of the self at collective and relational levels has distinct implications for individual motivation and behavior because it serves to redefine the nature of the self that is implicated in processes of self-actualization and self-enhancement (Turner, 1985; Haslam et al., 2000; Ellemers et al., 2004; Haslam, 2004; Sluss and Ashforth, 2008). Thus when group or role-based identities are salient, as is often the case in work contexts (Haslam, 2004), a person can be driven as much by a desire to enhance a collective sense of self (e.g., as a woman, a care worker, or an employee of a given organization) as they are by a desire to enhance their relational sense of self (e.g., in their role as supervisor or carer) or their personal sense of self (as a unique individual; e.g., Tim, Mary). In a range of circumstances this also means that acting in the interests of group membership can override concerns about personal self-interest (Onorato and Turner, 2001). As Ellemers et al. (2004, p. 461) assert “self-conception in collective terms would energize people to exert themselves on behalf of the group, facilitate the direction or effort toward collective (instead of individual) outcomes and help workers sustain their loyalty to the team or organization through times when this is not individually rewarding.”

Speaking to the value of this approach, a broad body of research has shown that organizational identification is positively related to a range of work-related attitudes and behaviors such as motivation, performance, job satisfaction, turnover intentions, and absenteeism (for reviews, see Tyler and Blader, 2000; Van Knippenberg, 2000; Haslam, 2004; Riketta and Van Dick, 2005). More recent organizational research on the strength of identification with different foci of attachment has also found that people typically indicate greater levels of identification with localized identities such as teams (Riketta and Van Dick, 2005; Riketta and Nienaber, 2007) and role relationships (Sluss et al., 2012; Smith et al., 2012) than with the organization as a whole. At the same time, organizational identification is often the strongest predictor of work-related outcomes including motivation and well-being (Sluss et al., 2012; Smith et al., 2012; for a general discussion of the relationship between social identification and well-being, see Haslam et al., 2009; Jetten et al., 2012). Accordingly, to the extent that their identification with different work relationships and work groups is congruent with, or nested within, organizational identification, then one would expect workers to be more motivated and more satisfied at work (Van Knippenberg, 2000; Haslam et al., 2003; Ellemers et al., 2004; Wegge et al., 2006; Riketta and Nienaber, 2007; Ashforth et al., 2008; Akerlof and Kranton, 2010). This is because, in instances of such perceived alignment, by advancing the organization the individual will see themselves to be enhancing aspects of the (collectively defined) self. On the other hand, when they experience incongruence between their role relationships and their team or organization, people are likely to be less motivated and experience compromised well-being in the form of greater frustration and stress (Haslam and Reicher, 2006).

According to Sluss and Ashforth (2008), congruence between relational and collective identities is likely to be strengthened by (1) the degree of task interdependence (a particularly high level of which is evident between frontline care workers and clients; Karlsson and Rydwik, 2013); and (2) the degree to which the relational partner is prototypical of the organization or the working context. In line with this reasoning, it seems likely that whether care workers indicate strong relational identification with their clients and whether their client and organizational identities converge will depend on the extent to which the care worker perceives their caring role with the client to be supported by the organization.

### The Present Research

To take into account the social and relational context in which care work takes place, the present research seeks to examine the motivation of care workers through the lens of social identity theorizing. In particular, it seeks to investigate the link between what incentivizes people to work in adult social care and their motivation and well-being. More specifically, it seeks to explore the role of financial incentives and social relationships in motivating care workers and driving positive work-related experiences (e.g., greater job satisfaction, reduced stress). Here, rather than dichotomising the ‘love’ and ‘money’ aspects of working in care we argue that they will form integrated elements of care workers’ motivation to the extent they serve to build meaningful work-based identification.

In addition, this study also examines how care workers’ motivations and well-being might be influenced by the two different working domains in which care workers predominately operate and by the process of professionalization, (i.e., undertaking qualifications). In line with recommendations about how to apply social identity analysis in the field (Haslam et al., 2003; Haslam, 2014) the study also focuses on those work identities that were identified as self-relevant by care workers in previous qualitative work in the present program (Bjerregaard et al., in press b) — namely client, care staff, care professional, and organization identities.

### Hypotheses

Based on the above reasoning, this study sought to test five main hypotheses:

• H1. Care workers’ social identification with different groups at work will be positively related to their work motivation and well-being (Ellemers et al., 2004). Specifically, we expect that carers’ work motivation and well-being — that is, their pride, job satisfaction, and stress, as well as their job attachment (turnover intentions and pro-professionalization) — will be predicted by their identification with (a) the people they care for (*client identification*; H1a) and (b) the care organization they work for (*organizational identification*; H1b). Moreover, although care workers are (c) likely to indicate higher levels of client identification than organizational identification (H1c); their (d) organizational identification is likely to be the more proximal predictor of motivation and well-being (H1d).
• H2. Care workers will primarily be incentivized to work in care because of their caring relationship with clients (H2a). However, the extent to which valuing relationships with clients leads to higher levels of motivation and well-being at work, will be mediated by identification with the organization (H2b). Moreover, this organizational identification is expected to be predicted by client identification (H2c).
• H3. Care workers will be less incentivized by pay than by other social considerations (H3a). Nevertheless, the extent to which pay does lead to increased motivation and well-being will also be mediated by organizational identification (H3b). Here the effects of organizational identity are unlikely to be predicted by client identification (H3c).
• H4. Carers’ sense of social identity will vary as a function of their place of work (i.e., residential/nursing home or domiciliary care). The nature of independent working involved in domiciliary care will lead workers to have higher levels of client identification (H4a) and lower levels of organizational identification (H4b) than those working in residential care. This lack of congruence between identities makes it more likely that domiciliary care workers will report lower levels of work motivation and well-being than those who work in residential care (H4c).
• H5. Undertaking a qualification is likely to lead to increased motivation and well-being (H5a). Again this is expected to be mediated by organizational identity (H5b). More specifically, the effects of undertaking a qualification on motivation and well-being should be explained by the extent to which undertaking a qualification increases and maintains identification with the organization and other groups at work (H5c).

### Study Context

To test these hypotheses, we administered an organizational survey to carers at two time points, 1 year apart. The surveys were distributed across multiple sites (in different locations across the south of England) in a large care organization that had recently amalgamated a number of smaller organizations. The survey measured carers’ motivation, their sense of identity at work, and their feelings about work outcomes, including professionalization. As well as allowing us to examine the relationship between these variables cross-sectionally, the study’s longitudinal design also enabled us to examine variation in responses over time as a function of whether or not people had undertaken professional qualifications in the intervening period—so that, in effect, undertaking a professional qualification in the past year constituted an experimental treatment (for similar logic, see Lim and Putnam, 2010). In this way, the study had a quasi-experimental longitudinal design, which enabled us to examine the impact of exposure to professional training on organizational identification and motivation.

## Materials and Methods

Surveys were administered to care staff who worked for a large not-for-profit organization that operates across the South of England and the Isle of Wight. The care organization, which recently incorporated four different care organizations, runs 28 residential care and nursing homes and delivers domiciliary care services from six domiciliary care bases. Its clients are primarily elderly people, yet services are also provided for younger people who need support to live independently on their own and in groups. The studies were conducted at two time points, 1 year apart, in January 2010 (T1) and January 2011 (T2).

### Participants

**Table 1** provides a detailed breakdown of sample characteristics. At Time 1, 3280 questionnaires were distributed and 643 were returned completed — a response rate of 20%. The majority of participants (*n* = 458; 72%) worked in residential and nursing care (residential care), 28% (*n* = 172) worked in domiciliary care. At Time 2, 4,200 questionnaires were distributed. Of these, 1274 completed questionnaires were returned — a 33% response rate. The majority of respondents (58%, *n* = 740) worked in residential care while 42% (*n* = 534) worked in domiciliary care. The substantially higher response rate at Time 2 was primarily the result of efforts to raise awareness of the survey among care staff through an article in the organization’s newsletter and presentations to managers. This had an especially notable effect in increasing the response rate of domiciliary care staff. A total of 204 carers participated on both occasions, 70% in residential care and 30% in domiciliary care. Of these, 51% (*n* = 103) had undertaken a professional qualification over the course of the year (i.e., were exposed to a professionalization treatment) and 49% (*n* = 100) had not.

**Table 1:** Sample demographics.

	Time 1	Time 2	Longitudinal
No. q’res distributed	3,280	4,200	
No. q’res returned	643	1,274	204
Response rate	20%	33%	
% Residential care	72%	58%	70%
% Domiciliary care	28%	42%	30%
Gender	*F* = 571 (92%) *M* = 51	*F* = 1077 (90%) *M* = 124	*F* = 188 (92%) *M* = 15
Age range	16–76 *M* = 44.8, *SD* = 12.12	16–78 *M* = 42.57, *SD* = 14.90	18–78 *M* = 46.62, *SD* = 14.27
**Job role**			
Domestic workers	52 (8.5%)	124 (10%)	20 (10%)
Care workers	364 (57%)	885 (69%)	124 (61%)
Snr care workers	115 (18%)	114 (9%)	27 (13%)
Managers	43 (6%)	51 (4%)	21 (10%)
Admin and planners	21 (3%)	36 (3%)	10 (5%)
Undisclosed role	48 (7.5%)	64 (5%)	2 (1%)

### Measures

Participants completed a four-page, 51-item questionnaire, in which they indicated agreement with statements on scales ranging from 1 (*strongly disagree*) to 7 (*strongly agree*). Work motivation was measured by means of five scales that examined (a) *job satisfaction* (three items, T1 α = 0.79, T2 α = 0.76, typical items: “I enjoy my work at [the care organization]”; after Haslam and Reicher, 2006); and (b) *pride* (three items, T1 α = 0.71, T2 α = 0.75, typical item: “*I am proud to work in the care sector*,” adapted from Tyler and Blader, 2000); (c) *stress* (four items, reversed scored, T1 α = 0.70, T2 α = 0.69; typical item: “I am able to cope with the demands of my job” adapted from Haslam and Reicher, 2006); (d) *turnover intentions* (two items, reversed scored, T1 *r* = 75, T2 *r* = 0.72, typical item: “I would like to stay working at [the care organization] for as long as possible”; after Ellemers et al., 1999); (e) *pro-professionalization* (five items, T1 α = 0.76, T2 α = 0.75, typical item: “I feel positive about the process of professionalization at [the care organization]”).

Key factors which *incentivize carers* to work for the care organization were measured by the extent to which (a) *pay* and (b) *relationship with clients* were valued. In each case, responses were given to a single item indicating whether “*I work at [the care organization] primarily because I value the [incentive]*.” At Time 2 this measure was supplemented by an item in which respondents indicated their *satisfaction with incentives* by responding to statements of the form: “Overall I am satisfied with the [incentive] at [the care organization]”: pay, *r* = 0.47, relationship with clients, *r* = 0.60.

*Work identification* was measured by four three-item scales that asked participants about different loci of identification in the workplace: (a) with clients (*client identification)*; (b) with staff at the care home or domiciliary base (*staff identification*); (c) with *care professionals (professional identification)*; and (d) with the organization *(organizational identification)*. For each measure respondents indicated whether “I feel strong ties with [group],” “I feel good about [group],” “I am willing to do as much as possible to make life easy for [group]” (adapted from Doosje et al., 1995; T1 αs = 0.78, 0.65, 0.65, 0.73, respectively; T2 αs = 0.70, 0.76, 0.72, 0.71, respectively).

At the end of the questionnaire participants were asked to provide demographic information (age, gender, length of service working, and ethnicity), as well as information about the nature of their work, their occupational role (domestic staff, care worker, senior care worker, manager, administrator or planner), and their work domain (residential and nursing vs. domiciliary care). At Time 2 participants were asked additional questions about the amount of training days they had undertaken in the past year, whether they had undertaken professional qualification in this time, and, if so, at what level.

### Ethical Approval

The research was approved by the School of Psychology Ethics Committee at the University of Exeter. This required participant anonymity and data confidentiality.

### Procedure

A questionnaire containing the above scales was distributed to care staff in sealed envelopes via the care homes and domiciliary bases. Prior to administration, the questionnaire was piloted on a small sample of 15 care workers in order to check and refine its terminology and structure. Questionnaires were accompanied by a cover letter from the organization and the researchers’ university that outlined the purpose of the survey and informed participants that completion of the questionnaire was taken as an indication of their consent to take part in the study, but that this was voluntary. Confidentiality and anonymity were assured. Respondents then returned the questionnaire in an enclosed stamped addressed envelope to the University. To enable questionnaires from the same person to be linked, respondents were requested to provide a unique identifying code. The recruitment of participants was intended to cast a wide net such that the sample was representative of all care workers working in domiciliary, residential and nursing care rather than any particular sub-sample.

## Results

### Analytic Strategy

Given the large number of respondents at T2 and the good representation of responses from domiciliary workers as well as residential care workers (that was missing from the T1 and longitudinal data), we decided to conduct our analysis on the cross-sectional T2 data and focused our analysis of longitudinal data on the changes that occurred over time, primarily around the impact of professionalization. Cross-sectional analysis proceeded in two steps. First, preliminary tests of hypotheses were conducted by examining bivariate correlation between the various measures administered in the study at each time — in particular, the relationships among (a) work group identification and motivation/well-being (H1), (b) perceived incentives and motivation/ well-being (H2, H3), (c) type of care work and identification (H4), and (d) professionalization activity (undertaking a qualification) and motivation/well-being (H5).

Second, we used structural equation modeling (SEM) to test two integrated theoretical models. Model 1 examined whether different incentives to work in care, namely relationships with clients and pay, are associated with greater motivation and well-being to the extent that they build relational identification with clients (H2a) and through this contribute to broader organizational identity (H3a, H3b). Model 2 then considered how the care work context — in this case, care workers’ work domain and their experience of having undertaken a qualification — was associated with motivation and well-being at work, and whether any effects could be accounted for by strengthened organizational identification (H4c and H5b).

A second phase of analysis was conducted on the longitudinal data (*n* = 204) and examined variation in responses over time as a function of whether or not respondents had undertaken a qualification in the intervening period. In this analysis, having undertaken qualifications during the intervening period (1 year) was treated as a quasi-experimental intervention. This enabled us to assess whether undertaking a qualification had any impact on identification and, through this, motivation and well-being (H5c).

### Cross-sectional Analysis

#### Bivariate Associations

Mean, standard deviation, and bivariate correlation for cross-sectional data at T2 are reported in **Table 2**. As can be seen from this table, participants generally reported positive work experiences and outcomes: they indicated very high levels of satisfaction, high levels of pride, and low levels of stress. They also reported being attached to their job, as reflected in low turnover intentions and favorable attitudes toward professionalization. As in other studies of the social care workforce (e.g., Heyes, 2005), carers attached the least value to pay and highest value to their relationships with clients as incentives for work.

**Table 2:** Bivariate correlation, Time 2.

	*M*	*SD*	1a	1b	2a	2b	3a	3b	3c	3d	4a	4b	4c	4d
1a Qualification taken	1.51	0.50												
1b Working domain	1.43	0.50	-0.08^∗^											
**Work incentive**														
2a Pay	3.10	1.46	0.02	0.03										
2b Relationship with clients	6.02	0.81	0.07^∗^	0.07^∗^	0.02									
**Work identification**														
3a Client	5.92	0.77	0.04	0.05	0.06^∗^	0.64^∗∗^								
3b Staff	5.37	0.95	0.10^∗∗^	0.09^∗∗^	0.15^∗∗^	0.31^∗∗^	0.46^∗∗^							
3c Organization	5.21	0.99	0.10^∗∗^	0.12^∗∗^	0.31^∗∗^	0.34^∗∗^	0.45^∗∗^	0.63^∗∗^						
3d Care professional	5.29	0.92	0.11^∗∗^	0.14^∗∗^	0.17^∗∗^	0.34^∗∗^	0.52^∗∗^	0.73^∗∗^	0.62^∗∗^					
**Work motivation and well-being**														
4a Satisfaction	6.02	0.87	0.05	0.04	0.23^∗∗^	0.37^∗∗^	0.43^∗∗^	0.43^∗∗^	0.53^∗∗^	0.425^∗∗^				
4b Pride	5.68	0.97	0.08^∗^	0.01	0.16^∗∗^	0.40^∗∗^	0.44^∗∗^	0.38^∗∗^	0.46^∗∗^	0.42^∗∗^	0.50^∗∗^			
4c Stress	2.40	0.85	-0.05	-0.01	0.18^∗∗^	0.20^∗∗^	0.31^∗∗^	0.41^∗∗^	0.43^∗∗^	-0.36^∗∗^	0.57^∗∗^	0.34^∗∗^		
4d Professionalization	5.46	0.87	0.10^∗∗^	0.10^∗∗^	0.15^∗∗^	0.26^∗∗^	0.34^∗∗^	0.41^∗∗^	0.51^∗∗^	0.39^∗∗^	0.47^∗∗^	0.42^∗∗^	0.49^∗∗^	
4e Turnover	2.51	1.40	-0.05	0.10^∗∗^	0.23^∗∗^	0.26^∗∗^	0.30^∗∗^	0.43^∗∗^	0.57^∗∗^	-0.39^∗∗^	0.56^∗∗^	0.40^∗∗^	0.43^∗∗^	0.42^∗∗^

*^∗∗^*p* < 0.01 (two-tailed), ^∗^*p* < 0.05 (two-tailed).*

In line with H1, participants indicated strongest identification with clients. They also identified strongly with the other staff where they worked, with care professionals in general, and with the organization itself (all mean > 5 on a seven-point scale). As predicted by H1, and as demonstrated in the bivariate correlation, higher identification with different groups at work (clients, organization, staff and care professionals) was associated with higher levels of well-being, a positive orientation to professionalization, and lower turnover intentions. Inspection of the bivariate correlation also revealed a clear pattern whereby the strength of association between identification and well-being and job attachment increased as the locus of identification became inclusive (i.e., higher-level and broader) rather than exclusive (lower level and narrower). Thus, at the exclusive end of the spectrum, relational identification with clients was positively associated with satisfaction, pride and more positive attitudes toward professionalization (*r*s = 0.43, 0.44, 0.34, respectively; all *p*s < 0.01) and was negatively associated with stress and turnover intentions (*r*s = -0.32, -0.34, respectively; all *p*s < 0.01). However, at inclusive end of the spectrum these relationships were all stronger — such that collective identification with the organization was strongly positively correlated with satisfaction, pride, and positive attitudes toward professionalization (*r*s = 0.53, 0.46, 0.51, respectively; all *p*s < 0.01) and was strongly negatively correlated with stress and turnover intentions (*r*s = -0.41, -0.61, respectively; all *p*s < 0.01).

Bivariate correlation also pointed to a variable degree of association between different incentives and workers’ motivation. In line with H2 and H3, these ranged from weak correlation between motivation, well-being and being incentivised by pay, to very strong correlation between motivation, well-being and being incentivised by relationships with client. More specifically, being incentivised by pay was weakly associated with increased satisfaction, pride, pro-professionalization attitudes as well as reduced stress and lower turnover intentions (*r*s = 0.23, 0.16, 0.15, -0.18, -0.23, respectively; all *p*s < 0.01). However, there were stronger associations between being incentivised by relationships with clients and motivation and well-being in terms satisfaction, pride, pro-professionalization attitudes, reduced stress, and reduced turnover intentions (*r*s = 0.37, 0.40, 0.26, -0.20, -0.26, respectively; all *p*s < 0.01).

Consistent with H4a, undertaking domiciliary (vs. residential) care work was generally associated with stronger client identification and weaker colleague, professional, and organizational identification. Consistent with H4c there were also negative associations between domiciliary care work and motivation, reflected in attitudes that were less pro-professionalization and stronger turnover intentions. However, there were no significant correlation between type of care work and measures of satisfaction, pride, and stress.

Finally, and consistent with H5, undertaking a qualification was positively associated with increased staff team identity, care professional identity, and organizational identity. In addition, undertaking a qualification was positively associated with key measures of motivation and well-being— notably increased pride and pro-professionalization. In sum, these patterns of association reveal relationships that are broadly consistent with our hypotheses. However, to explore these patterns of support in more detail, we conducted SEM.

#### Structural Equation Modeling

**FIGURE 1:**
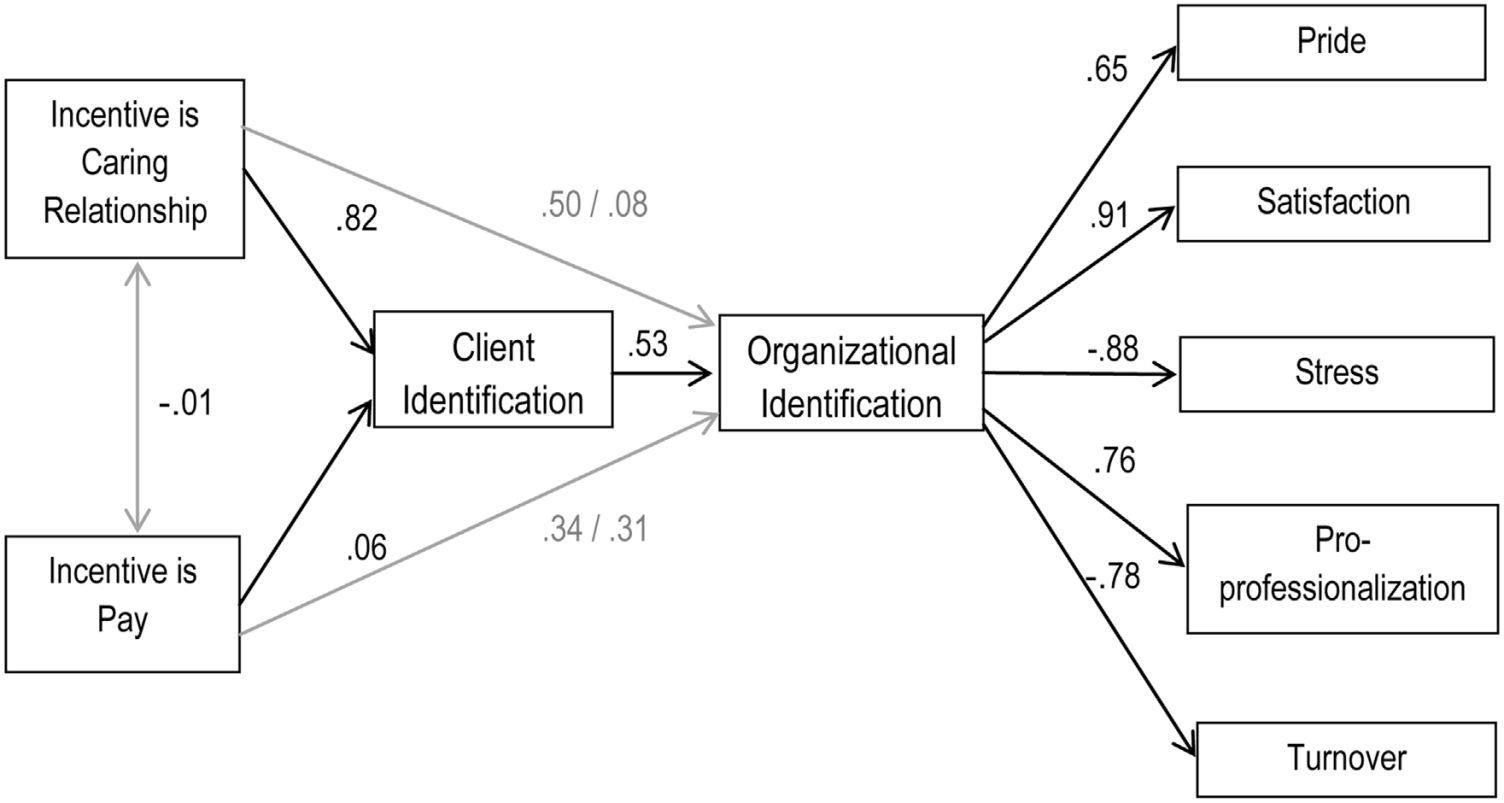
**The effects of incentive source on motivation and well-being (Model 1, Time 2)**.

**FIGURE 2:**
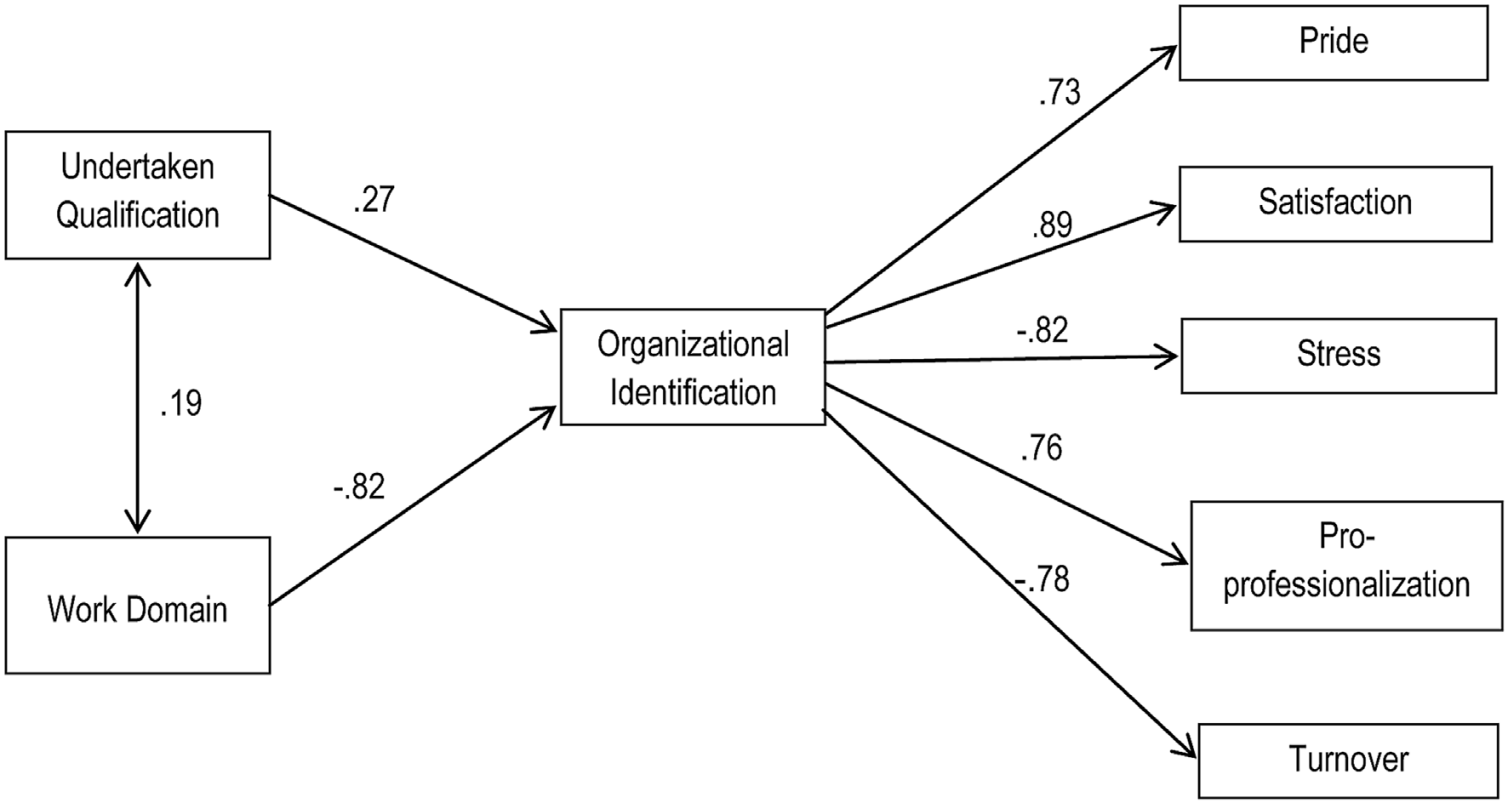
**The effects of undertaking a qualification and work domain (residential vs. domiciliary) on motivation and well-being (Model 2, Time 2)**.

Two theoretical models were tested by SEM using AMOS 19 software. Model 1, presented in **Figure 1**, examined the relationship between what care workers reported incentivizes them to work in care and their motivation and well-being at work, and the way in which this is influenced by identification with clients and the organization. Model 2, presented in **Figure 2**, examined whether work domain and having undertaken a qualification were related to work motivation and well-being and whether any effects that were observed were mediated by the degree to which these things strengthened (or undermined) organizational identification.

##### Model 1

Two sets of SEM results are reported to test Model 1: (a) the results of confirmatory factor analysis which establishes whether indicators measure the corresponding latent variables within the model and (b) the fit of the relationships outlined in the model between latent variables (following McDonald and Ho, 2002; Garson, 2009; Hayes, 2012). To determine the fit of the proposed models we report three Goodness-of Fit indices (as suggested by Garson, 2009): the chi-square χ^2^ (where values below 5 indicate an acceptable fit, values below 2 indicate a good fit); one incremental fit index, the Comparative Fit Index (CFI; where indices range from 0 to 1, with values exceeding 0.90 indicating a good fit); and one residual fit index, the root mean squared error of approximation (RMSEA), which is based on the proportion of variance not explained in the model (where values above 0.08 indicate poor fit, above 0.05 indicate good fit, and below 0.05 indicate excellent fit; see Hu and Bentler, 1999, for further discussion of the appropriate application of Goodness-of-Fit indices to test Model fit; see also Garson, 2009; Kenny, 2012).

In order to provide additional support for our models, we test a null model and compare our models to plausible alternative models, which are outlined in greater detail below. The null model (where all the parameters are set to zero) tests the assumption that there is no co-variation among the variables that make up the model and provides a baseline against which to compare the theoretical model (Crabtree et al., 2010). In addition to testing this model on the largest, most representational sample (the cross-sectional data at Time 2) we further corroborated our proposed theoretical model by conducting the analysis on the longitudinal data (*n* = 204).

Confirmatory Factor Analysis validated the measurement of the model, establishing that the indicators (i.e., the items) in the model measured the corresponding latent variables (i.e., the measures), χ^2^(360) = 4.78, CFI = 0.92, RMSEA = 0.055. As expected, the null model did not fit the data well, with a highly significant chi-square indicating a significant difference between the observed and estimated covariance matrices, χ^2^(392) = 38.94, CFI = 0.001, RMSEA = 0.18.

The hypothesized model then tested our integrated theory (a) that the effects of incentives on organizational identity are mediated by identification with clients (H1) and (b) that what incentivises people to work in care (relationships with clients or pay) leads to enhanced motivation and well-being outcomes because it builds identification with the organization (H2 and H3). The unadjusted theoretical model fitted the data well, c*^2^*(392) = 5.90, CFI = 0.89, RMSEA = 0.060. In line with common practice we also examined how the fit of the model might be improved by inspecting the standardized residual matrix for highly correlated error terms and then allowing these to correlate in the model (Ullman, 1996; Crabtree et al., 2010). Highly correlated error terms were observed among indicators within a number of latent variables (organizational identification, client identification, satisfaction, stress, and pro-professionalization) and so these were allowed to correlate. This adjusted theoretical model also had a good fit to the data, $\eta_{p}^{2}$(405) = 4.55, CFI = 0.92, RMSEA = 0.054. In short, the application of this model to the data confirms its robust fit.

The fit of the adjusted theoretical model was also compared to plausible alternative models. Alternative Model A turned the hypothesized model around and examined the possibility that participants’ motivation and well-being explains what it is that incentivizes them to work in care and that this is mediated by their level of client and then organizational identification. This had very poor fit to the data, c*^2^*(392) = 14.89, CFI = 0.69, RMSEA = 0.11. Alternative Model B examines a traditional economic model of motivation which suggests that people are incentivised to work in care as a function of their levels of motivation without any mediating role for processes of identification. This also had poor fit, c*^2^*(392) = 13.40, CFI = 0.73, RMSEA = 0.10. Alternative Model C examined the possibility that organizational identification leads to client identification, and that the latter mediates the relationship between factors that incentivize participants to work in care and levels of motivation and well-being. Although it had better fit, this did not fit the data as well as the theoretical model, c*^2^*(392) = 6.05, CFI = 0.87, RMSEA = 0.064. In summary, our theoretical model appears to provide a better representation of our data than other models that propose plausible alternative casual sequences.

##### Model 2

Structural equation modeling was also used to test theoretical Model 2 — that different working domains and the process of undertaking qualifications would affect motivation and well-being to the extent that they serve to build organizational identification. This followed the same analytical logic as Model 1 above. Here, confirmatory factor analysis validated the measurement of the model and, as expected, the null model did not fit the data well, as evidenced by a highly significant chi-square, c*^2^*(392) = 43.64, CFI < 0.001, RMSEA = 0.186.

The theoretical model examined the relationship between having undertaken qualifications in the last year, and subsequent motivation, and the extent to which these relationships were mediated through identification with the organization. This fitted the data substantially better than the null model and, after adjusting the model to allow a number of highly correlated items to correlate (organizational identification, satisfaction, and pro-professionalization), the amended theoretical model had good fit to the data, c*^2^*(156) = 4.92, CFI = 0.92, RMSEA = 0.056. In line with recommended best practice, to test the robustness of the model we repeated it on a subsample of data comprised of those participants who took part at both times (*n* = 203). This also provided evidence of good fit.

We also tested two other plausible alternative theoretical models. Alternative Model A tested the possibility that client identification predicted organizational identification, and that both of these sequentially mediated the relationships between undertaking a qualification and working domain on the one hand and increasing well-being and motivation on the other. This model had a good fit with the data, c*^2^*(156) = 5.15, CFI = 0.90, RMSEA = 0.058, but not as good as the theoretical model. A second plausible alternative, Model B, assessed whether undertaking a qualification and working domain led directly to increased motivation and well-being. This model had poor fit, c*^2^*(156) = 10.90, CFI = 0.82, RMSEA = 0.090.

### Longitudinal Analysis: Variation in Responses as a Function of Undertaking a Professional Qualification

To look at the effect of time on participants’ motivation we conducted an examination of the longitudinal data across T1 and T2 (*N* = 204)^3^. We started by conducting a paired samples *t*-test on measures of motivation and identification at the two times. In general, participants’ responses were highly consistent across the year. Nonetheless, (a) participants’ level of commitment rose over the course of the year, *t*(197) = 2.72, *p* < 0.005, (b) their attitude toward professionalization became more positive *t*(186) = 3.20, *p* < 0.005, (c) their identification with care professionals decreased, *t*(191) = 1.96, *p* < 0.05, (d) the value they attached to working conditions rose, *t*(199) = 2.02, *p* < 0.05, as did (e) the value they attached to opportunities for training and development, *t*(198) = -2.53, *p* < 0.05.

**FIGURE 3:**
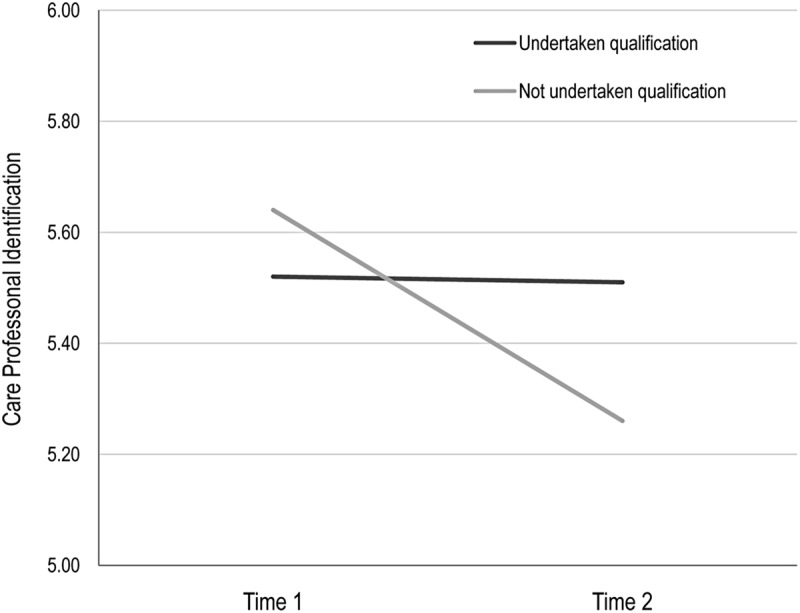
**The effects of undertaking a qualification on care professional identification, Longitudinal panel data**.

Against this backdrop of evidence that there was little change in motivation and identification over the course of the year, and taking into account the pathway analysis tested in the SEM models (which demonstrated that motivational and well-being outcomes were proceeded by identification with work groups), we then tested the effects of undertaking professional qualification on identification (i.e., H5c). To this end, panel data analysis enabled us to refine our investigation and control for reverse causality and selection bias (following the strategy employed by Lim and Putnam, 2010). Here, participants’ identification with care professionals was examined by mean of a 2 (Professionalization: gained vs. did not gain qualification) × 2 (Phase: Time 1 vs. Time 2) ANOVA, with repeated measures on the second factor. This analysis revealed a significant effect for phase, Wilks Lambda = 0.96, *F*(1,201) = 7.37, *p* = 0.007, $\eta_{p}^{2}$ = 0.04, indicating that, as noted above, participants were less identified with care professionals at T2 (*M* = 5.39, *SD* = 1.0) than at T1, (*M* = 5.51, *SD* = 0.81). However, this effect was conditioned by a significant interaction between professionalization and phase, Wilks Lambda = 0.97, *F*(1,201) = 6.51, *p* = 0.01, $\eta_{p}^{2}$ = 0.03. This interaction is plotted in **Figure 3**. Tests for simple effects revealed that there was no difference over time in the care professional identification of participants who had taken part in training between T1 (*M* = 5.52, *SD* = 0.81) and T2 (*M* = 5.51, *SD* = 0.97) Wilks Lambda 1.00, *F*(1,201) = 0.02, *p* = 0.91, $\eta_{p}^{2}$ = 0.00. However, there was a significant reduction in care professional identification among those who had not undertaken qualifications across these two time points (T1 *M* = 5.64, *SD* = 0.08; T2 *M* = 5.26, *SD* = 0.09), Wilks Lambda = 0.94, *F*(1,201) = 14.08, *p* < 0.001, $\eta_{p}^{2}$ = 0.06.

**FIGURE 4:**
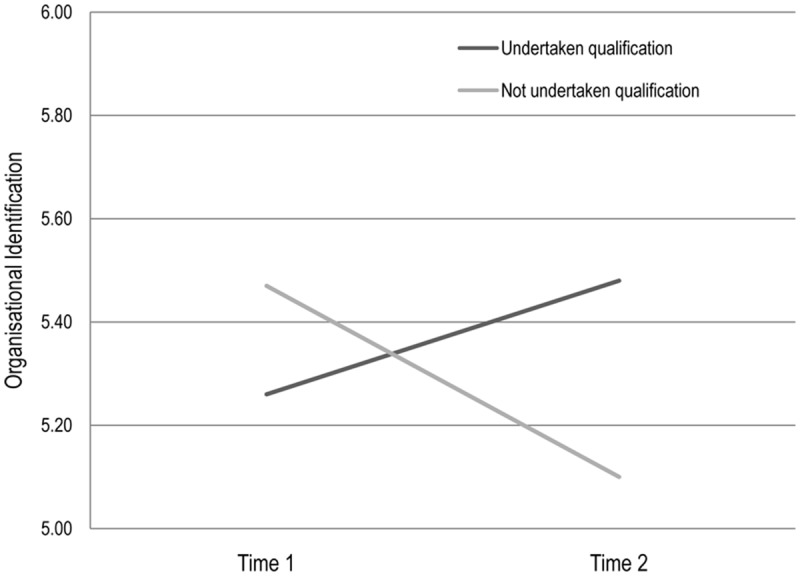
**The effects of undertaking a qualification on organizational identification, Longitudinal panel data**.

The same analysis was performed for the measure of participants’ identification with the organization. This revealed no main effect for phase [Wilks Lambda = 0.99, *F*(1,201) = 0.10, *p* = 0.75] but a significant interaction between professionalization and phase, Wilks Lambda = 0.97, *F*(1,201) = 3.83, *p* = 0.03, $\eta_{p}^{2}$ = 0.02. This interaction is plotted in **Figure 4**. Tests for simple effects revealed that the organizational identification of participants who had undertaken a qualification rose, albeit not significantly, from Time 1 (*M* = 5.26, *SD* = 0.09) to Time 2 (*M* = 5.48, *SD* = 0.10), Wilks Lambda = 0.98, *F*(1,201) = 3.1, *p* = 0.08, $\eta_{p}^{2}$ = 0.02, but that the organizational identification of those who had not undertaken qualifications decreased, again not significantly, from at Time 1 (*M* = 5.47, *SD* = 0.11) to Time 2 (*M* = 3.1, *SD* = 0.10), Wilk Lambda = 0.99, *F*(1,201) = 1.78. *p* = 0.18, $\eta_{p}^{2}$ = 0.009.

The same analysis was also performed on participants’ identification with clients. This revealed a similar pattern to that observed with care profession identification, however, there was no significant main effect for phase [Wilks Lambda = 0.99, *F*(1,201) = 1.18, *p* = 0.28] and no significant interaction effect between group and phase [Wilks Lambda = 0.99, *F*(1,201) = 2.21, *p* = 0.14].

In summary, our findings show that engaging in the professionalization process by acquiring a qualification served to increase carers’ identification with the organization, and to maintain their identification with care professionals, but that not engaging in this process was associated with a reduction in both forms of identification. This accords with evidence from our pathway analysis where Model 2 suggests that undertaking a qualification leads to increased motivation and well-being outcomes because it serves to build organizational identification.

## Discussion

The purpose of this study was to examine how care-workers’ motivation and well-being is shaped by their social and relational identification at work. More specifically, the study sought to gain a better understanding of the ways in which care workers’ motivation and well-being are structured (a) by material (pay) or virtuous (relationships with clients) reward, (b) by the working context (of residential vs. domiciliary care), and (c) by exposure to professionalization (through undertaking vs. not undertaking a qualification). It also sought to investigate the role of identity processes in accounting for the effects of these factors on the process of creating and sustaining compassionate work cultures in care organizations.

Based on a social identity theorizing, we argued that organizational identification provides a critical underpinning for sustainable motivation and well-being in the workplace (Ellemers et al., 2004; Jetten et al., 2012). Consequently, factors that serve to build or reinforce organizational identification (e.g., incentives or specific forms of training) should have a positive impact on individual motivations and well-being at work. At the same time, this analysis was supplemented by the proposal (gleaned from qualitative research; Bjerregaard et al., in press b) that for this particular workforce (and arguably many other ‘helping’ occupations) relational identification with clients, patients, or service users is central to the way in which workers define themselves within the broader organizational context. This suggestion also accords with previous research in the social identity tradition (Sluss and Ashforth, 2008; Sluss et al., 2012), which suggests that motivation and well-being should be particularly enhanced when these different bases of identification — relational and organizational — align. Accordingly, we also examined the relationships between relational and organizational identifications and their combined role in supporting carer motivation and well-being.

### Summary of Findings

Consistent with other studies undertaken with care workers (Skills for Care, 2007; Hussein et al., 2010; Atkinson and Lucas, 2012) participants in our study reported high levels of satisfaction and pride, and low levels of stress. Participants were motivated to work in care because of their relationships with clients, and they attached high value to training and personnel development, and to working conditions, but relatively low value to pay. In line with previous work (Van Dick, 2001; Haslam et al., 2003; Van Knippenberg et al., 2004; Riketta and Van Dick, 2005) and with H1, findings also showed that care workers’ identification with different groups at work is positively related to their motivation and well-being. In particular, motivation and well-being were predicted both by identification with clients (H1a) and by organizational identification (H1b). As anticipated, care workers indicated higher levels of identification with clients than with the organization (H1c), but levels of motivation and well-being were best predicted by the latter (H1d).

### Effects of Incentives

Findings from SEM confirmed that although care workers were primarily incentivised by ‘love’ (i.e., relationships with clients) rather than ‘money’ (i.e., pay), attaching value to either of these incentives was associated with increased motivation and well-being (H2a, H3a). Importantly, and consistent with our hypotheses, these relationships were also mediated by patterns of identification (H2b, H3b). Specifically, being incentivised by relationships with clients fed into motivation and well-being by increasing relational identification and, through this, organizational identification (H2c). A similar pattern was evident for incentivization by pay; however, here the mediating role of organizational identification was stronger (H3c).

Contrary to mainstream economic thinking about care work, these findings suggest that neither altruism nor money in themselves lead to more positive organizational outcomes. Rather, they contribute to enhanced motivation and well-being due largely to their ability to foster and reinforce care workers’ organizational identification. Thus, rather than being particularly valued in its own right, pay can be seen to play an important role in motivating staff and making them feel good about themselves and their work because it helps build organizational identification — for example, by indicating to the individual that they are valued by the organization (Tyler and Blader, 2000). Likewise, enacting relationships with clients promotes motivation and well-being because this behavior reflects the way in which individuals see themselves within the organization. Indeed, the very weak fit of a model that represented the mainstream economic and individualistic perspectives — wherein incentives directly affect motivation and well-being — suggests that this oft-cited dichotomised explanation for work in this domain fails properly to explain key outcomes in this domain.

### Effects of Work Domain

In line with H4, care workers’ attachment to their job (i.e., a positive orientation to professionalization and low turnover intentions) was found to vary as a function of their working domain such that residential care workers displayed generally higher organizational motivation than domiciliary workers. Again, though, SEM indicated that these domain-based differences in motivation could again be accounted for by patterns of identification (H4b and 4c) in so far as the best fitting model was one in which differences in organizational identification mediated the relationship between work domain and motivation and well-being.

### Effects of Professionalization

In line with other research into the effects of identification on training outcomes (Pidd, 2004; Bjerregaard et al., in press a), our findings indicate that undertaking a qualification increases well-being and attachment to one’s job. However, consistent with our overall theoretical framework, these effects of training — which we were able to examine longitudinally — could also be understood in terms of their consequences for organizational identification. Specifically, it appears that undertaking a qualification increased well-being and attachment because this reinforced individuals’ sense of identification with their organization (H5a). Interestingly, although we predicted that organizational identification would be most important for explaining the effects of gaining a qualification, an alternative model that incorporated client identification as a precursor to organizational identification accounted for the data nearly as well. This pattern mirrors the pattern observed for incentivization and further underscores the important linkages between client-based and organizational identification in this domain.

### Theoretical Implications: Identity Convergence and the Creation of a Compassionate Workforce

These findings corroborate and extend findings from previous organizational studies that have pointed to the positive mediating effects of organizational identification on motivational and well-being outcomes (see Van Knippenberg, 2000; Haslam et al., 2003; Ellemers et al., 2004; Ashforth et al., 2008; Jetten et al., 2012; van Dick and Haslam, 2012). In addition, this research considered the dynamic relationships between organizational identity and other work-based identities that operate at different levels of abstraction (Riketta and Nienaber, 2007; Sluss et al., 2012; Smith et al., 2012). In particular, we explored the relationship between relational identification with clients (or service users/patients) and organizational identification and found that client identification can become a basis for organizational identification, and accordingly that the forces that strengthen the former can also strengthen the latter — with positive consequences for different aspects of organizational motivation and well-being.

Tests of alternative models that considered reversed sequences of mediators demonstrated poorer fit to the data, suggesting that although there is convergence between different forms of identification (i.e., relational and organizational), it is likely to be the former that builds the latter rather than the other way around. This may reflect the fact that because client identification operates at a relational level of identity it is “a linchpin in overall self-concept at work” (Sluss and Ashforth, 2008, p. 11). Drawing on findings from our previous qualitative work with care workers (Bjerregaard et al., in press b), this convergence between the specific relational identification and broader organizational identification is likely to occur through two processes. First, it may occur by means of ‘affect transfer,’ whereby “affects generated from identifying with a role relationship directly and unconsciously transfer to the organization and vice versa” (Sluss and Ashforth, 2008, p. 5). That is, the positive affect generated from identifying with the carer role is transferred to the assessment of the organization in terms of its capability to care (both for clients and carers themselves; Bjerregaard et al., in press b). Second, it may also occur through the process of “behavioral sense-making” whereby “what one does, informs and confirms who one is” (Sluss and Ashforth, 2008, p. 6). That is, enacting a carer role that one identifies with informs how one thinks both about oneself as a representative of the organization and about the organization as a whole.

In these ways, this study supports the notion that the different levels of care-worker identification (relational identification with clients and social identification with the organization) converge around care workers’ perceptions that the organization values (a) their relationship with their clients, (b) the interests of the clients, and (c) the care workers themselves. At the same time it explains care workers’ disengagement and frustration at the organization should they perceive it not to be acting in accordance with caring values. Accordingly, at least in the context of care work, it could be argued that the creation of a compassionate working culture depends upon the organization’s capacity to both (a) to harness and support meaningful identities through which individuals understand their work and (b) to promote alignment between work-based identities at all levels (personal, relational, and organizational).

### Practical Implications

At a practical level, this study helps us to see how the social identity approach can provide a multi-dimensional, dynamic framework through which to better understand care workers’ motivation and well-being. First, by integrating the virtuous and financial aspects of caring — that is, by seeing both of these as reward that underpin workplace identities — this model challenges the simplistic view that care workers’ motives are dichotomised between either ‘love’ or ‘money.’ Instead, it appears that care workers are motivated and buoyed by both ‘love’ of the caring relationships they have with their clients and ‘money’ as reflective of the caring relationship that exists between them and the organization for which they work. Second, this framework suggests that in order to be successful in building a compassionate culture in health and social care, attention needs to be paid to people’s prevalent work-based relational and social identities (e.g., as argued by Haslam et al., 2003; Peters et al., 2013).

Amongst other things, these identities are created in conversations that draw on social, cultural, organizational and individual narratives, and which let people know who they are and why they matter (Harquail and King, 2010; Ibarra and Barbulescu, 2010; Bjerregaard, 2015). Accordingly, the values and behaviors that are attributed to these different identities can make them congruent and mutually supportive (e.g., seeing oneself to be both a caring professional and member of a caring organization) or can make them ambiguous, irrelevant and conflicting (e.g., seeing oneself as part of a family while at the same time being made to feel like a number and a commodity; Bjerregaard et al., in press b). The degree of alignment between multiple meaningful social identities in and out of work should also have consequences for sustained employee motivation. Hence, third, undertaking activities which support professionalization and embed compassionate practice are likely to motivate care workers and to make them feel good about both themselves and their work to the extent that these activities harness and develop meaningful organizational and relational identities that enable those workers to experience a congruence between the values and behaviors represented by different foci of social identification at work.

## Conclusion

This study provides detailed cross-sectional and longitudinal evidence of the way in which care workers’ motivation is shaped by the dynamics of collective and relational identities at work. In this context, the application of the social identity approach has two key affordances. First, it allows us to understand the nuanced ways in which care workers are motivated and satisfied by both ‘love’ and ‘money.’ Second, it shows how these two things feed into increased motivation and well-being by building and maintaining meaningful work-based identities, in particular organizational identity. It thus appears that to the extent that particular incentives validate social identities at work, they will serve to make individuals more motivated to engage with their work and to feel better (both about themselves and their work) when they do. Conversely, when incentives negate valued identities, this is likely to result in detrimental outcomes for both the individual and the organization.

In arriving at this conclusion, we believe the present research goes some way to addressing calls to “examine the team and organizational influences that contribute to compassionate care and determine how they can be strengthened” (Adamson et al., 2012, p. 32). It does so by confirming that compassionate behavior and values are enacted and sustained by care workers to the extent they make sense in terms of their salient social and relational identities. Accordingly, it appears that it is by recognizing, harnessing, and developing these identities that care organizations can encourage those they employ to play their part in enacting a culture of compassionate care.

## Conflict of Interest Statement

The authors declare that the research was conducted in the absence of any commercial or financial relationships that could be construed as a potential conflict of interest.
